# Singular adaptations in the carbon assimilation mechanism of the polyextremophile cyanobacterium *Chroococcidiopsis thermalis*

**DOI:** 10.1007/s11120-023-01008-y

**Published:** 2023-03-20

**Authors:** Pere Aguiló-Nicolau, Jeroni Galmés, Giacomo Fais, Sebastià Capó-Bauçà, Giacomo Cao, Concepción Iñiguez

**Affiliations:** 1 Research Group on Plant Biology Under Mediterranean Conditions, Universitat de les Illes Balears, INAGEA, Ctra. Valldemossa km. 7.5, 07122 Palma, Balearic Islands Spain; 2 Interdepartmental Centre of Environmental Science and Engineering, University of Cagliari, Via San Giorgio 12, 09124 Cagliari, Italy; 3 Department of Mechanical, Chemical and Materials Engineering, University of Cagliari, Via Marengo 2, 09123 Cagliari, Italy

**Keywords:** Cyanobacteria, Rubisco, CO_2_-concentrating mechanisms, CO_2_-fixation, Photosynthesis

## Abstract

Cyanobacteria largely contribute to the biogeochemical carbon cycle fixing ~ 25% of the inorganic carbon on Earth. However, the carbon acquisition and assimilation mechanisms in Cyanobacteria are still underexplored regardless of being of great importance for shedding light on the origins of autotropism on Earth and providing new bioengineering tools for crop yield improvement. Here, we fully characterized these mechanisms from the polyextremophile cyanobacterium *Chroococcidiopsis thermalis* KOMAREK 1964/111 in comparison with the model cyanobacterial strain, *Synechococcus* sp. PCC6301. In particular, we analyzed the Rubisco kinetics along with the in vivo photosynthetic CO_2_ assimilation in response to external dissolved inorganic carbon, the effect of CO_2_ concentrating mechanism (CCM) inhibitors on net photosynthesis and the anatomical particularities of their carboxysomes when grown under either ambient air (0.04% CO_2_) or 2.5% CO_2_-enriched air. Our results show that Rubisco from *C. thermalis* possess the highest specificity factor and carboxylation efficiency ever reported for Cyanobacteria, which were accompanied by a highly effective CCM, concentrating CO_2_ around Rubisco more than 140-times the external CO_2_ levels, when grown under ambient CO_2_ conditions. Our findings provide new insights into the Rubisco kinetics of Cyanobacteria, suggesting that improved *S*_c/o_ values can still be compatible with a fast-catalyzing enzyme. The combination of Rubisco kinetics and CCM effectiveness in *C. thermalis* relative to other cyanobacterial species might indicate that the co-evolution between Rubisco and CCMs in Cyanobacteria is not as constrained as in other phylogenetic groups.

**Supplementary Information:**

The online version contains supplementary material available at 10.1007/s11120-023-01008-y.

## Introduction

Cyanobacteria is one of the most primitive life forms on Earth (Knoll 2008). With the appearance of oxygenic photosynthesis more than 2500 Mya, cyanobacterial metabolism changed the composition of the primitive atmosphere, rising oxygen levels with the consequent decrease in CO_2_ (Blank 2013; Schirrmeister et al. 2016; Sánchez-Baracaldo et al. 2022). Atmospheric oxygen rise permitted the evolution of a more complex life, leading to the large biological variability found in the present (Dismukes et al. 2001). Nowadays, the photosynthetic activity of Cyanobacteria makes an important contribution to the biosphere carbon cycle, with recent estimations indicating that it represents more than 25% of the total CO_2_ fixation on Earth (Rae et al. 2013). Cyanobacteria occupy a wide variety of habitats such as terrestrial, marine, brackish-water, freshwater, and even extreme environments (Tomitani et al. 2006). However, the carbon acquisition and assimilation mechanisms of the diverse cyanobacterial group have been barely studied, and research has only been focused on a few model cyanobacterial species (Espie and Kimber 2011; Whitehead et al. 2014; Xia et al. 2020; Basu and Mackey 2022).

The ultimate responsible of the inorganic carbon fixation in photosynthetic organisms is the enzyme Ribulose-1,5-bisphosphate carboxylase/oxygenase (Rubisco, EC 4.1.1.39) (Spreitzer and Salvucci 2002). Besides its carboxylating activity, Rubisco also catalyzes the oxygenation of Ribulose-1,5-bisphosphate (RuBP), leading to the photorespiration pathway, which consumes energy and leads to inorganic carbon loss (Hamilton 2019). Other two catalytic particularities of Rubisco are its relatively poor affinity for CO_2_ and low carboxylation turnover rate (${k}_{{{\mathrm{cat}}}}^{{\mathrm{c}}}$) (Galmés et al. 2014b). These catalytic limitations of Rubisco constrain the CO_2_ assimilation capacity of photosynthetic organisms and, under stressful conditions, it may even compromise achieving sufficient rates of inorganic carbon fixation to support autotropism (Bauwe et al. 2010, 2012). Three different adaptative mechanisms have been described to occur in photosynthetic organisms that allow them to counterbalance Rubisco catalytic limitations: (i) increasing Rubisco concentration, (ii) increasing CO_2_ concentration at the active sites of Rubisco, and (iii) optimizing Rubisco kinetics to the intracellular concentrations of the two gaseous substrates, CO_2_ and O_2_ (Flamholz and Shih 2020).

Rubisco constitutes the most abundant enzyme on Earth, being up to 50% of the total soluble protein in C_3_ plant leaves (Ellis 1979; Spreitzer and Salvucci 2002). Nevertheless, there is a large variability in Rubisco content among photosynthetic organisms, which is linked to nutrient (especially nitrogen) and CO_2_ availability (Andersson and Backlund 2008). For example, higher amounts of the enzyme have been found in organisms that depend on the diffusive CO_2_ entry from the atmosphere to the sites of carboxylation (Raven 2013). On the contrary, other organisms evolved mechanisms that increase CO_2_ around Rubisco active sites, i.e., the so-called CO_2_ concentrating mechanisms (CCMs), which lead to a lower Rubisco content (Losh et al. 2013). One example of the latter is Cyanobacteria, where Rubisco only accounts for 2 to 10% of the total soluble protein (Dai et al. 2018). The organisms presenting CCMs are C_4_ and CAM terrestrial vascular plants, seagrasses, algae, Cyanobacteria, and some proteobacteria (Iñiguez et al. 2020; Capó-Bauçà et al. 2022b). Two types of CCMs have been described, biochemical and biophysical. The former involves a CO_2_ fixation prior to that catalyzed by Rubisco (C_4_ and CAM plants), and the latter involves the active transport of HCO_3_^−^/CO_2_ across membranes and/or an increase in the external CO_2_ concentration by acidification of the extracellular environment (aquatic organisms, i.e.: seagrasses, algae, Cyanobacteria, and Proteobacteria; Giordano et al. 2005). Cyanobacteria and some proteobacteria have evolved a particular CCM component consisting of a proteic polyhedral shell filled with Rubisco and carbonic anhydrase (CA), called carboxysome (Whitehead et al. 2014). HCO_3_^−^ is actively accumulated in the cytosol, where there is no CA activity, and enters the carboxysome, where CA catalyzes the dehydration of HCO_3_^−^ to CO_2_ and increases the CO_2_ concentration around Rubisco, therefore enhancing carboxylation over oxygenation (Mangan and Brenner 2014; Mangan et al. 2016). This prokaryotic type of CCM is one of the most efficient inorganic carbon acquisition mechanisms, concentrating CO_2_ around Rubisco active sites up to 100 times the extracellular CO_2_ levels (Badger and Andrews 1987).

Regarding the optimization of Rubisco kinetics, a large variability in the main kinetic parameters has been observed across photosynthetic organisms (Young et al. 2016; Bathellier et al. 2018; Flamholz et al. 2019; Iñiguez et al. 2020). The highest values of CO_2_/O_2_ specificity (*S*_c/o_; up to 240 mol mol^−1^) and the highest affinities for CO_2_ (which means the lowest Michaelis–Menten semi-saturation constant for CO_2_ measured at 0% O_2_, *K*_c_; down to 3.3 µM) are found in Rhodophyta (Whitney et al. 2001)*,* whereas the highest Rubisco carboxylation turnover rates ($k_{{{\mathrm{cat}}}}^{{\mathrm{c}}}$) are found in Proteobacteria (up to 22.2 s^−1^, Davidi et al. 2020). However, the vast majority of the Rubisco kinetic data to date belongs to higher plants, implying an important bias towards other phylogenetic groups (Flamholz et al. 2019; Iñiguez et al. 2020). Large variability in Rubisco kinetics has been observed in the few cyanobacterial strains analyzed so far, with *S*_c/o_ values ranging between 32 and 60 mol mol^−1^; *K*_c_ ranging between 80 and 309 µM, the Michaelis–Menten semi-saturation constant for O_2_ (*K*_o_) ranging between 529 and 1400 µM, and $k_{{{\mathrm{cat}}}}^{{\mathrm{c}}}$ ranging between 2.41 and 14.4 s^−1^ (Iñiguez et al. 2020). By contrast, in higher plants, *S*_c/o_ values range from 60 to 120 mol mol^−1^, *K*_c_ from 6 to 44 µM, *K*_o_ from 150 to 1500 µM, and $k_{{{\mathrm{cat}}}}^{{\mathrm{c}}}$ from 1 to 7 s^−1^.

The three variables described above that determine the carbon fixation capacity of an autotrophic organism (Rubisco concentration, CO_2_ concentration at the sites of Rubisco carboxylation, and Rubisco kinetics) are not independent of each other, and it is believed that they have co-evolved shaped by both phylogeny and environment (Galmés et al. 2014a; Tcherkez et al. 2018). For example, the presence of CCMs in terrestrial vascular plants is correlated with a lower Rubisco content and an enhancement of $k_{{{\mathrm{cat}}}}^{{\mathrm{c}}}$ at the expense of a loss in Rubisco affinity for CO_2_ (higher *K*_c_) (Galmés et al. 2014b). However, the co-evolution of Rubisco kinetics and CCMs have only been widely investigated in higher plants, leaving other phylogenetic groups, such as Cyanobacteria, understudied. In addition, extremophile organisms present specific adaptations to optimize carbon fixation under unfavorable conditions; therefore, one question that remains to be answered is whether the analysis of extremophile cyanobacteria could widen the range of variability of Rubisco kinetics and CCM operation found in the previously analyzed model species.

To answer if extremophile cyanobacteria could possess singular adaptations in the carbon acquisition and assimilation mechanisms, we performed a complete analysis on Rubisco kinetics, operation of inorganic carbon acquisition mechanisms, CCM effectiveness and anatomical imaging of *Chroococcidiopsis thermalis* KOMAREK 1964/111, a polyextremophile cyanobacterium inhabiting desertic rock surfaces, supporting temperatures up to 50 °C, high UV radiation and desiccation (Billi et al. 2011; Cumbers and Rothschild 2014); in comparison with the model species *Synechococcus* sp. PCC6301. We hypothesize that *C. thermalis* has evolved multiple mechanisms that allowed its adaptation to a wide range of CO_2_ conditions derived from the harsh environments where this cyanobacterium inhabits.

## Materials and methods

### Cyanobacterial cultures

Cyanobacterial strains of *Synechococcus* sp. (PCC 6301/UTEX 625; CCALA 188) and *Chroococcidiopsis thermalis* (KOMAREK 1964/111; CCALA 048) were acquired from the Culture Collection of Autotrophic Organisms (CCALA, Třeboň, Czech Republic). *Synechococcus* sp. was grown in 1 L sterilized flasks under orbital agitation, and *C. thermalis* was grown in 140 mm × 20 mm glass Petri dishes without agitation. Both cultures were maintained in Z-medium (Staub 1961) at 20 °C in a 16:8 light–dark cycle with a light intensity of 50 μmol m^−2^ s^−1^ for *Synechococcus* sp. and 30 μmol m^−2^ s^−1^ for *C. thermalis* (according to their optimum growth irradiances) provided by a cool-white light source (4000 K; Osram L 18W/840 Lumilux, Germany), in a temperature-controlled chamber (Aralab Fitoclima S600 PLH, Spain). Growth rate was followed by spectrophotometry at 650 nm (OD_650_, Thermo scientific Multiskan Sky 1530-00433C, USA). When cultures reached an OD650 value of 0.4, control and enriched CO_2_ experiments were started, each with three autoclaved flasks of 100 mL culture connected to a constant air-flux of 5 mL/min of either ambient air (0.04% CO_2_, LC) or 2.5% CO_2_-enriched air (HC), respectively. Both species were grown under constant agitation during the experiment and maintained in exponential growth phase. After 7–10 days of acclimation to the two CO_2_ treatments, the physiological measurements described below were done.

### O_2_ evolution measurements

Net photosynthesis and the effect of external and internal carbonic anhydrases (CAs) and anion-exchange bicarbonate transporter inhibitors on net photosynthesis were determined at the culture temperature (20 °C) by monitoring O_2_ evolution using Clark-type oxygen electrode chambers (Oxygraph, Hansatech, UK). 2 mL of culture were placed in the chamber, illuminated with white-light LED lamps at a saturating photosynthetic irradiance [300 μmol m^−2^ s^−1^, previously determined for both species by chlorophyll *a* fluorescence light curves using a pulse-amplitude-modulated fluorometer (Dual-PAM-100, Walz, Germany)]. O_2_ evolution rates were taken at 2–3 min intervals after rate stabilization, using the O2view software (version 2.10, Hansatech). Rates were normalized to the dry weight (DW) of biomass, which was obtained weighing the dried 2 mL pellet of each measurement.

To assess the role of CAs in photosynthesis, the percentage of net photosynthesis inhibition after adding 200 µM of acetazolamide (AZ, external CAs inhibitor) and 200 µM of ethoxyzolamide (EZ, external and internal CAs inhibitor) was monitored. The same procedure was followed after adding 300 µM of the anion-exchange transporter inhibitor 4,4′-diisothiocyanatostilbene-2,2′-disulfonate (DIDS) to a fresh 2 mL culture aliquot.

Photosynthesis-dissolved inorganic carbon (DIC) curves were done at saturating photosynthetic irradiance (300 μmol m^−2^ s^−1^) at 25 °C to be able to compare the obtained in vivo carbon fixation rates with those from in vitro Rubisco measurements (also done at the standard temperature of 25 °C). 2 mL fresh culture was washed three times with CO_2_ free-Z medium with 20 mM Tris–HCl (pH 8), by gentle centrifugation (3000×*g* for 3 min). Initial O_2_ concentration inside the chambers was lowered to 70% by bubbling with N_2_ to avoid O_2_ oversaturation during the curve. Oxygen saturation in air-equilibrated media was determined using DOTABLES (https://water.usgs.gov/software/DOTABLES/) software for the specific conductivity of the medium at 25 °C. After zero net photosynthesis was detected, increasing concentrations of DIC were added every 2–3 min, obtaining rates for 8–12 different DIC concentrations in the chamber (0–1500 µM for LC-grown cells and 0–5000 µM for HC-grown cells). Dissolved CO_2_ concentration in equilibrium in the medium for each DIC concentration assayed was calculated using CO2sys software and curves were fitted to the Michaelis–Menten equation obtaining the maximum photosynthetic rate (*A*_max_) and the in vivo photosynthetic semi-saturation constant for CO_2_ (*K*_m in vivo_).

### Carbon isotopic discrimination

The ^13^C isotopic discrimination in cyanobacterial cells was obtained from a 50 mL culture aliquot at OD_650_ of 0.8–1, which was centrifuged at 10,000×*g* for 3 min. The pellet was freeze-dried overnight and homogenized. 0.2 g of the dried powder was transferred into metallic capsules (176980926, Lüdiswiss, Switzerland) and combusted in an elemental analyzer (Thermo Flash EA 1112 Series, Germany) where CO_2_ was injected into a continuous-flow isotope mass spectrometer (Thermo-Finnigan Delta XP, Bremen, Germany). Peach leaf standards (NIST 1547) were measured every six samples. Results are presented as δ vs. PDB (Pee Dee Belemnite). The obtained ^13^C isotopic discrimination of the cyanobacterial biomass (δ^13^C) was corrected with the ^13^C isotopic composition of DIC found in the medium from either CO_2_-enriched or control experiments, as described by Iñiguez et al. (2016).

### Total soluble proteins and Rubisco quantification

50 mL culture aliquots at OD_650_ of 0.8–1 were centrifuged at 10,000×*g* for 3 min. Pellets were inmediately frozen in liquid nitrogen and homogenized in a mixer mill (Retsch GmbH MM200) with 2 mL cold Extraction Buffer containing 100 mM EPPS (pH 8.1), 1 mM ethylenediaminetetraacetic acid (EDTA), 20 mM MgCl_2_, 2% CelLytic™ B (B7435, Sigma-Aldrich), 1 M dithiothreitol (DTT), 2% plant protease inhibitor cocktail (P9599, Merck, USA), 100 mM ${\upbeta }$-mercaptoethanol and 0.1 g polyvinylpolypyrrolidone (PVPP). The homogenate was then centrifuged for 5 min at 3000×*g* at 4 °C. The supernatant was kept on ice and the pellet was frozen in liquid nitrogen and milled again. This process was repeated three times to ensure maximum extraction efficiency.

A supernatant aliquot was used to quantify the total soluble protein (TSP) content following Bradford’s (1976) method, and to quantify Rubisco content by Western blot immunodetection of the Rubisco large subunit using purified Rubisco standard and Rubisco large subunit antibody (AS01 017S and AS03 037 Agrisera, Sweden) at 1:20,000 dilution and Goat anti-Rabbit IgG HRP-conjugated secondary antibody (AS09 602 Agrisera, Sweden) at 1:50,000 dilution (see Supplementary Fig. 2).

### Rubisco catalytic measurements

The previously described crude protein extract was partially purified using a 5 mL Mini-Macroprep High-Q strong anion-exchange cartridge (Bio-Scale Mini Macro-Prep High Q Cartridge 7324124, Bio-Rad, USA) and then desalted and concentrated ~ tenfold using Amicon Ultra 4 (Z740198, Merck, USA) by centrifuging at 1000×*g* at 4 °C. Rubisco carboxylation kinetic traits were determined at 25 °C as explained in (Capó-Bauçà et al. 2020) 7 mL-septum capped crystal vials with magnetic stirrer containing 400 µL of Assay Buffer (100 mM Bicine (pH 8.1), 20 mM MgCl_2_) and ~ 100 W-A units of carbonic anhydrase (C3934 Merck, USA) were bubbled with 100% N_2_ gas or CO_2_-free synthetic air (21% O_2_, 79% N_2_) for 2 h. After that, one of eight different concentrations of NaH^14^CO_3_ from 0 to 60 mM with a specific activity of 3.7 × 10^10^ Bq mol^−1^ and 1.6 mM of RuBP [synthesized and purified as explained in Kane et al. (1994)] were added to each vial. The semi-purified protein extracts were supplemented with 20 mM NaH^14^CO_3_ and pre-activated for 30 min at 35 °C (optimum incubation time and temperature for full Rubisco activation, as previously determined). Assays were started by adding 20 µL of preactivated extract and led to react for 1 min (final reaction volume of 0.495 mL). The reaction was stopped by adding 200 µL of 10 M formic acid and dried at 80 °C. Non-volatile acid-stable ^14^C-organic molecules were determined by scintillation counting (Beckman Coulter LS6500, USA).

The semi-saturation constant for CO_2_ under 0% and 21% O_2_ (*K*_c_ and $K_{{\mathrm{c}}}^{{21{\text{ \% O}}_{2} }}$, respectively) and the maximum carboxylation velocity ($V_{{{\mathrm{max}}}}^{{\mathrm{c}}}$) were determined from fitting the data to the Michaelis–Menten equation. The semi-saturation constant for O_2_ (*K*_o_) was calculated in each biological replicate by a linear fit of *K*_c_ measurements obtained under 0% and 21% O_2_. CO_2_ concentration in solution was calculated assuming a carbonic acid dissociation constant (pK_a_) of 6.11 at 25 °C (Galmés et al. 2016) using accurate measurements of the assay buffer pH at 25 °C. $k_{{{\mathrm{cat}}}}^{{\mathrm{c}}}$ was calculated by dividing $V_{{{\mathrm{max}}}}^{{\mathrm{c}}}$ by Rubisco active site’s concentration, the latter determined by incubating the same semi-purified protein extracts for 30 min at room temperature with 2ʹ-carboxyarabinitol-1,5-bisphosphate (^14^C-CABP) (Ruuska et al. 1998), assuming eight active binding sites per Rubisco (Blayney et al. 2011). The optimal concentration of ^14^C-CABP for Rubisco quantification was determined for each cyanobacterial strain as explained in (Capó-Bauçà et al. 2022b).

Rubisco’s specificity factor (*S*_c/o_) was assayed with [1-^3^H]RuBP as explained by Kane et al. (1994), using the same semi-purified extracts as for kinetics. 7 mL septum-capped crystal vials containing 940 µL of Assay buffer (30 mM Triethanolamine-acetate (pH 8.3) and 15 mM Mg-acetate), 400 W-A units of carbonic anhydrase and 20 µL of semi-purified protein extract were bubbled with a gas mixture of 99.95% O_2_ and 0.05% CO_2_ for 1 h. The reaction was initiated by adding ~ 1 nmol of [1-^3^H] RuBP and incubated for 1 h at 25 °C with continuous stirring (final reaction volume of 1 mL). The reaction was stopped by adding 0.35 U of alkaline phosphatase (P7640, Merck, USA). The reaction product was purified using anion exchange AG1-X8 resin (1401441, Bio-Rad, USA), and then, glycolate and glycerate picks were separated by high-performance liquid chromatography (HPLC; Jasco-UV-4075, Jasco inc., USA) and quantified by scintillation counting (Beckman Coulter LS6500, USA). *S*_c/o_ was calculated using a CO_2_/O_2_ solubility ratio of 0.038 at 25 °C.

Rubisco kinetic parameters obtained in the present study for *Synechococcus* sp. and *C. thermalis* were compared to all cyanobacterial Rubisco kinetic data available to date. These data were extracted from the compilation of Iñiguez et al. (2020), which includes Rubisco kinetic data from phylogenetically distant organisms (see Supplementary Spreadsheet 1).

### Transmission electron microscope imaging

1 mL culture aliquots (OD_650_ ~ 0.8) were centrifuged at 10,000×*g*. The pellet was then resuspended in 1 mL fixation buffer (0.1 M phosphate buffer pH 7.2, 4% glutaraldehyde, 2% paraformaldehyde) and stored at 4 °C under darkness. Post-fixation was performed in 1% osmium tetroxide, prepared in 0.1 M Sorensen’s phosphate buffer, for 1 h. The fixed sections were then stained in 2% uranyl acetate, dehydrated in a graded ethanol series, and embedded in London Resin White (EMS, Hatfield, PA). Semithin (1 µm thick) and ultrathin (50 to 70 nm thick) sections were cut using an ultramicrotome (UC7/FC7; Leica, Germany). The semithin sections were mounted on glass slides and stained with epoxy tissue stain (EMS, Hatfield, PA). The ultrathin sections were mounted on copper grids and visualized using the transmission electron microscope Jeol JEM 1400 operating at 80 kV. Image analysis was done using ImageJ software (Wayne Rasband National Institutes of Health, version 2.3.0/1.53q).

### Rubisco gross assimilation modeling

An adaptation of Farquhar’s biochemical model (Farquhar et al. 1980) was applied to the in vitro measured Rubisco kinetic traits to calculate the Rubisco gross assimilation rate per catalytic site (*A*_Rub_) at varying CO_2_ partial pressure at the Rubisco active sites of *Synechococcus* sp. and *C. thermalis* in comparison with the Rubisco kinetic traits of a model C_3_ crop species, *Triticum aestivum*, obtained from Iñiguez et al. (2020) (Eq. 1).

1 $$A_{{{\mathrm{Rub}}}} = \frac{{\left( {C - \Gamma^{*} } \right) \cdot k_{{{\mathrm{cat}}}}^{{\mathrm{c}}} \cdot {\mathrm{RCS}}}}{{C + K_{{\mathrm{c}}} \cdot \left( {1 + \frac{O}{{K_{{\mathrm{o}}} }}} \right)}}$$

where $k_{{{\mathrm{cat}}}}^{{\mathrm{c}}}$, *K*_c_ and *K*_o_ are the carboxylation turnover rate and the Michaelis–Menten semi-saturation constants for carboxylation and oxygenation, respectively, measured in vitro at 25 °C. C and O are the CO_2_ and O_2_ partial pressure at the Rubisco active sites which, in the case of CO_2_, range between 10 and 20,000 ppm, and, in the case of O_2_, partial pressure is assumed to be constant at 210,000 ppm. Γ^*^ = 0.5 O/*S*_c/o_, where *S*_c/o_ is the CO_2_/O_2_ specificity factor measured in vitro at 25 °C. RCS is the number of Rubisco catalytic sites which was set to 1 to obtain the Rubisco gross assimilation rate per catalytic site.

### Statistical analysis

The significance of differences was tested using two-way ANOVA after normality (Anderson–Darling test) and homoscedasticity (Levene test) was corroborated. For data that do not meet normality and/or homoscedasticity, the Kruskal–Wallis test was used to test the significance of differences. Post hoc comparisons were done using the Tukey test or the Bonferroni correction, respectively. Student’s *t* test, or Mann–Whitney–Wilcoxon test for non-parametric data, was used to compare means between two groups of data. *P* values below 0.05 were considered significant. Data were analyzed using R (version 3.2.3 and RStudio version 0.99.879) and plots were done using the ggPlot2 package (version 2.2.1).

## Results

### The singularity of Rubisco kinetics from *C. thermalis* among Cyanobacteria species

**Fig. 1:**
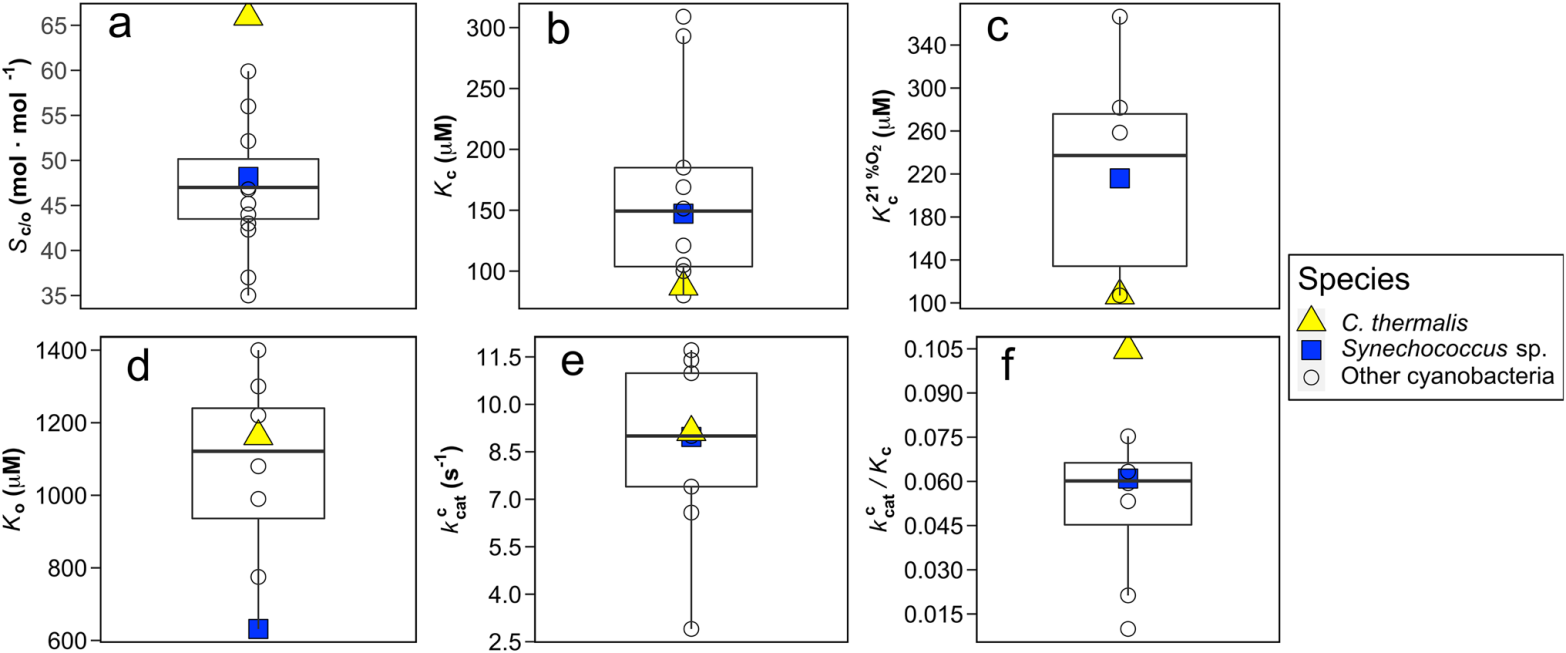
In vitro Rubisco kinetic traits at 25 °C: **a** CO_2_/O_2_ specificity factor (*S*_c/o_); **b** Michaelis–Menten semi-saturation constant for CO_2_ at 0% O_2_ (*K*_c_); **c** Michaelis–Menten semi-saturation constant for CO_2_ at 21% O_2_ ($K_{{\mathrm{c}}}^{{21{\text{ \% O}}_{2} }}$); **d** Michaelis–Menten semi-saturation constant for O_2_ (*K*_o_); **e** carboxylation turnover rate ($k_{{{\mathrm{cat}}}}^{{\mathrm{c}}}$), and **f** Rubisco carboxylation efficiency ($k_{{{\mathrm{cat}}}}^{{\mathrm{c}}}$/*K*_c_) of *Chroococcidiopsis thermalis* KOMAREK 1964/111 (yellow triangles) and *Synechococcus* sp*.* PCC6301 (blue squares), measured from semi-purified protein extracts of both strains in the present study, compared to Rubisco kinetics of other previously measured cyanobacterial strains (data compilation from Iñiguez et al. 2020, empty circles). 3–6 replicates of each Rubisco kinetic parameter were used to calculate the mean value shown for *Synechococcus* sp. PCC6301 and *C. thermalis* KOMAREK 1964/111 in the boxplots (mean values and standard deviations are shown in Supplementary Table 1). For the other cyanobacterial strains, kinetic parameters are the mean of all values reported in the compiled studies for each strain (values and references provided in Supplementary Spreadsheet 1)

In vitro Rubisco kinetic parameters measured at 25 °C show significant differences between *Synechococcus* sp. and *C. thermalis*, except for $k_{{{\mathrm{cat}}}}^{{\mathrm{c}}}$ (Supplementary Table 1). *Chrooococcidiopsis thermalis* presented 40% higher *S*_c/o_, 70% higher affinity for CO_2_ (i.e. lower *K*_c_), 85% lower affinity for O_2_ (i.e. higher *K*_o_), and 75% higher catalytic carboxylation efficiency ($k_{{{\mathrm{cat}}}}^{{\mathrm{c}}} /K_{{\mathrm{c}}}$) than *Synechococcus* sp. In addition, when compared with previously measured cyanobacterial Rubisco, *C. thermalis* possessed the most extreme values for most of the Rubisco kinetic parameters (Fig. 1). The highest value ever reported for *S*_c/o_ in Cyanobacteria corresponded to *C. thermalis* (69.0 mol mol^−1^) from the present study, being 1.4-fold higher than the cyanobacterial average (46.6 mol mol^−1^) and twofold higher than the lowest values, observed in *Anabaena* sp. PCC7120 (35.0 mol mol^−1^). The lowest values for *K*_c_ were found in *Aphanocapsa virescens* (Jordan and Ogren 1983) followed by *C. thermalis* (80.0 µM and 87.2 µM, respectively), both presenting the highest affinity for CO_2_ among all measured cyanobacteria. This Rubisco affinity for CO_2_ from *C. thermalis* was twofold higher than the cyanobacterial average, which means a twofold lower *K*_c_ than the average value of 167.78 µM for Cyanobacteria. When measured at 21% O_2_, the Michaelis–Menten semi-saturation constant for CO_2_ ($K_{{\mathrm{c}}}^{{21{\text{ \% O}}_{2} }}$) of *C. thermalis* was also the lowest among previously measured cyanobacteria (106.9 µM), followed by the hyperthermophile *Thermosynechococcus elongatus* BP-1 (107 µM; Wilson et al. 2018). This means that Rubisco CO_2_ affinity under 21% O_2_ in *C. thermalis* is more than twofold higher than the cyanobacterial average ($K_{{\mathrm{c}}}^{{21{\text{ \% O}}_{2} }}$ of 245.9 µM). Similar values of $k_{{{\mathrm{cat}}}}^{{\mathrm{c}}}$ were obtained for *Synechococcus* sp. and *C. thermalis* (9.0 s^−1^ and 9.1 s^−1^, respectively) that fit the cyanobacterial average ($k_{{{\mathrm{cat}}}}^{{\mathrm{c}}}$ of 9.8 s^−1^). In addition, the highest value of $k_{{{\mathrm{cat}}}}^{{\mathrm{c}}} /K_{{\mathrm{c}}}$ ever reported for Cyanobacteria was found again in *C. thermalis* (0.10 s^−1^ µM), being more than twofold higher than the cyanobacterial average (0.05 s^−1^ µM). The lowest affinity for O_2_ was observed in *Prochlorococcus marinus* MIT9313 (*K*_o_ of 1400 µM; Shih et al. 2016), whereas *C. thermalis* presented a relatively low affinity for O_2_, with a *K*_o_ of 1163 µM.

### Characterization and effectiveness of CO_2_ concentrating mechanisms in ***Synechococcus*** sp. and ***C. thermalis***

#### Net photosynthesis and effect of CCM inhibitors

**Fig. 2:**
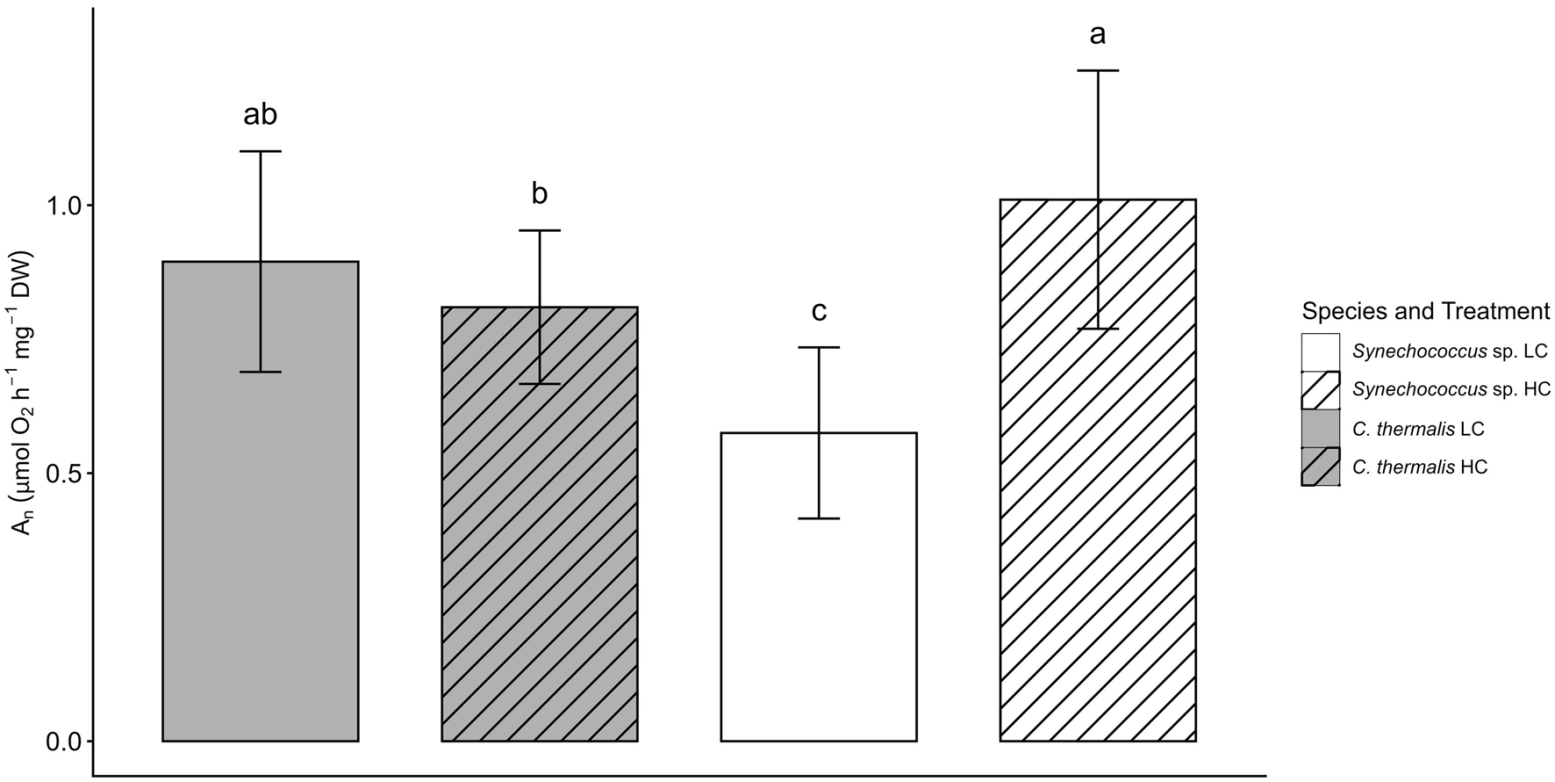
Net photosynthetic rate (*A*_*n*_) in *Synechococcus* sp. PCC6301 (white) and *Chroococcidiopsis thermalis* KOMAREK 1964/111 (grey) at 20 °C under ambient air (0.04% CO_2_, LC, empty pattern) or 2.5% CO_2_—enriched air (HC, line pattern) and saturating irradiance (300 μmol photons m^−1^ s^−1^). Values are means ± standard deviation of 10 replicates. Different letters denote significant differences among different strains and CO_2_ treatments (*P*<0.05, two-way ANOVA followed by Tukey’s test or Kruskal-Wallis test followed by Bonferroni correction for non-parametric data)

Net photosynthetic rate (*A*_*n*_) in *C. thermalis* was similar under both HC and LC, averaging ~0.9 µmol O_2_ h^–1^ mg^–1^ DW (Fig. 2). In contrast, *Synechococcus* sp. showed a ~two-fold higher *A*_*n*_ under HC than under LC, with *Synechococcus* sp. LC exhibiting the lowest *A*_*n*_ among all species-treatment combinations.

In *Synechococcus* sp., *A*_*n*_ was significantly inhibited by the addition of AZ (which only inhibits external CAs) under HC but not under LC, while in *C. thermalis,* *A*_*n*_ was inhibited by AZ in both CO_2_ treatments (Table 1). *Chroococcidiopsis thermalis* showed a higher percentage of inhibition by AZ under HC than *Synechococcus* sp. (35.4% and 23.5% of net photosynthetic inhibition, respectively). In addition, *A*_*n*_ was strongly inhibited by the addition of EZ (which inhibits both internal and external CAs) in both species and CO_2_ treatments (ranging from 47 to 87% inhibition), indicating the presence of constitutive internal CAs with an important contribution to net photosynthetic rate. The percentage of EZ inhibition in *C. thermalis* under HC was significantly higher than under LC (86.9% and 65.6%, respectively). No differences in the percentage of inhibition by EZ were observed between both species under LC, neither in *Synechococcus* sp. between HC and LC.

**Table 1:** Percentages of inhibition of net photosynthesis after the addition of the inhibitors acetazolamide (AZ), ethoxyzolamide (EZ) and 4,4′-diisothiocyanatostilbene-2,2′-disulfonate (DIDS) in *Synechococcus* sp*.* PCC6301 and *Chroococcidiopsis thermalis* KOMAREK 1964/111, both grown under ambient air (0.04% CO_2_, LC) or 2.5% CO_2_—enriched air (HC). Values are means ± standard deviations of 10 replicates

Species	CO_2_ treatment	% inhibition AZ	% inhibition EZ	% inhibition DIDS
*Synechococcus* sp.	LC	11.7 ± 5.9 a	51.4 ± 6.7 ab*^#^	20.2 ± 8.1 a*
*Synechococcus* sp.	HC	23.5 ± 9.3 b*	46.8 ± 12.1 b*^#^	44.3 ± 7.4 b*
*C. thermalis*	LC	20.3 ± 5.0 ab*	65.6 ± 14.9 a*^#^	6.1 ± 2.3 c
*C. thermalis*	HC	35.4 ± 10.7 c*	86.9 ± 17.7 c*^#^	19.3 ± 5.6 a*

Different letters denote significant differences among strains and CO_2_ treatments (*P* < 0.05, two-way ANOVA followed by Tukey’s test or Kruskal–Wallis test followed with Bonferroni correction for non-parametric data). Asterisk (*) indicates a significant inhibition of the net photosynthetic rate (*P* < 0.05, Student’s *t* test or Mann–Whitney–Wilcoxon test for non-parametric data). Hash (#) indicates significant differences between the net photosynthetic rate under AZ and that under EZ (*P* < 0.05, Student’s *t* test or Mann–Whitney–Wilcoxon test for non-parametric data)

*A*_*n*_ of the two species in both CO_2_ treatments was significantly inhibited by the addition of the anion-exchange transporter inhibitor DIDS, except for *C. thermalis* under LC (Table 1). *A*_*n*_ from *Synechococcus* sp. under HC was inhibited more than twofold by DIDS, in comparison with *C. thermalis* under HC (44.3 *vs.* 19.3% of inhibition). In addition, the percentage of DIDS inhibition in *Synechococcus* sp. under HC was significantly higher than under LC.

#### In vivo photosynthetic affinity for CO_2_ and CCM effectiveness

**Fig. 3:**
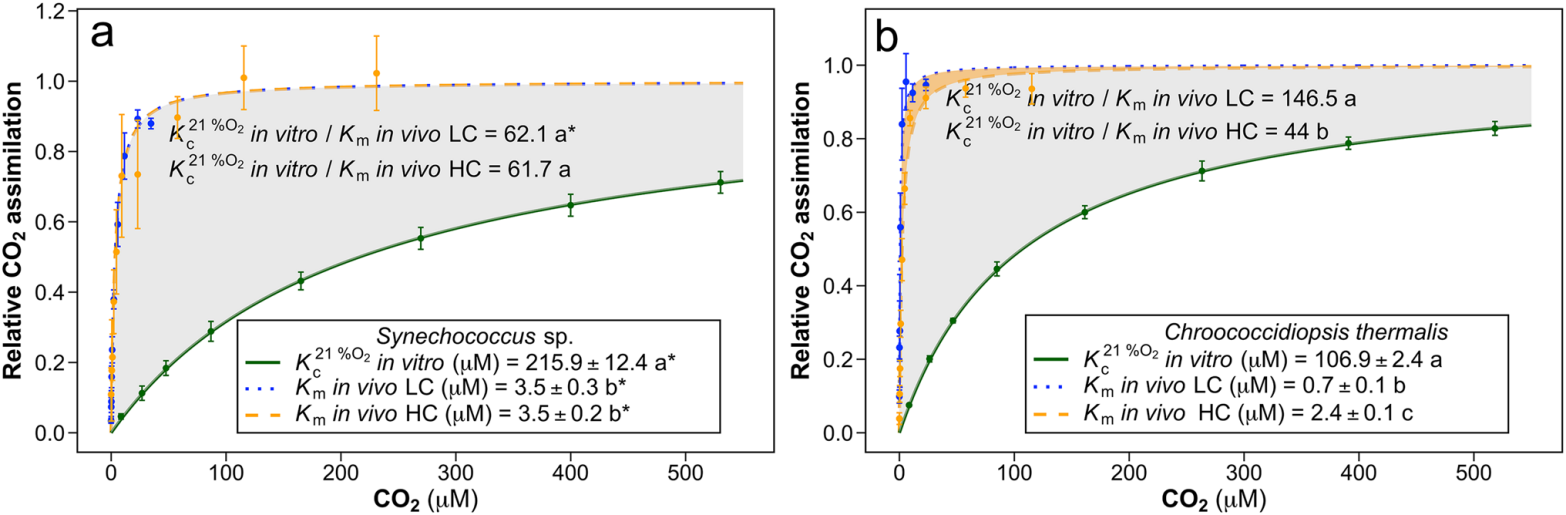
Rubisco in vitro CO_2_ assimilation under 21% O_2_ (green line), photosynthetic in vivo CO_2_ assimilation of ambient air grown cells (blue dotted line; LC) and photosynthetic in vivo CO_2_ assimilation of 2.5% CO_2_ grown cells (orange dashed line, HC) from **a** *Synechococcus* sp. PCC6301 and **b** *Chroococcidiopsisthermalis* KOMAREK 1964/111. The maximum Rubisco and photosynthetic CO_2_ assimilation rates were standardized to 1 in both plots. The ratio between the Rubisco in vitro Michaelis–Menten semi-saturation constant for CO_2_ under 21% O_2_ $(K_{{\mathrm{c}}}^{{21{\text{ \% O}}_{2} }} )$ and the photosynthetic in vivo Michaelis–Menten semi-saturation constant for CO_2_ from either cells grown under ambient air (*K*_m__in vivo_ LC) or cells grown under 2.5% CO_2_ (*K*_m__in vivo_ HC) indicates the CCM effectiveness. Different letters denote significant differences among treatments, and the asterisk (*) indicates significant differences between the two analyzed species (*P* < 0.05, Kruskal–Wallis test followed by Bonferroni correction for $(K_{{\mathrm{c}}}^{{21{\text{ \% O}}_{2} }} )$, *K*_m__in vivo_ LC and *K*_m__in vivo_ HC within species; and Student’s *t* test for parametric data, or Mann–Whitney–Wilcoxon test for non-parametric data, to compare means between species). 3–6 replicates were used to calculate the mean values of the Rubisco in vitro measurements and 10 replicates were used for the photosynthetic in vivo measurements

When comparing the two species grown under LC, *C. thermalis* presented a fivefold higher in vivo photosynthetic affinity for CO_2_ than *Synechococcus* sp. (i.e. fivefold lower *K*_m__in vivo_, with values of 0.7 µM and 3.5 µM, respectively; Fig. 3). *Synechococcus* sp. did not change its *K*_m__in vivo_ between LC and HC-grown cells, whereas *C. thermalis* presented a more than threefold lower in vivo photosynthetic affinity for CO_2_ when grown under HC than under LC (*K*_m__in vivo_ of 2.4 and 0.7 µM, respectively).

The effectiveness to concentrate CO_2_ around Rubisco active sites from the CCM machinery was assessed by comparing the in vivo photosynthetic response to CO_2_ with the in vitro Rubisco fixation response to CO_2_ under 21% O_2_ (Fig. 3 and Supplementary Fig. 1), through the ratio between $(K_{{\mathrm{c}}}^{{21{\text{ \% O}}_{2} }} )$ and $K_{{\mathrm{m}}}$_in vivo_. CCM effectiveness in *C. thermalis* grown under LC was more than twofold higher than that found in *Synechococcus* sp. grown under LC ($K_{{\mathrm{c}}}^{{21{\text{ \% O}}_{2} }}  /K_{{\mathrm{m}}}$_in vivo_ ratio of 146.5 µM µM^−1^ and 62.1 µM µM^−1^, respectively; Fig. 3). CCM effectiveness in *Synechococcus* sp. remained unvaried between the two CO_2_ treatments. By contrast, CCM effectiveness in *C. thermalis* grown under HC was more than threefold lower than that found under LC (ratio $K_{{\mathrm{c}}}^{{21{\text{ \% O}}_{2} }}  /K_{{\mathrm{m}}}$_in vivo_ ratio of 44 µM µM^−1^ and 146.5 µM µM^−1^, respectively).

#### Total Rubisco content and carbon isotopic fractionation

**Fig. 4:**
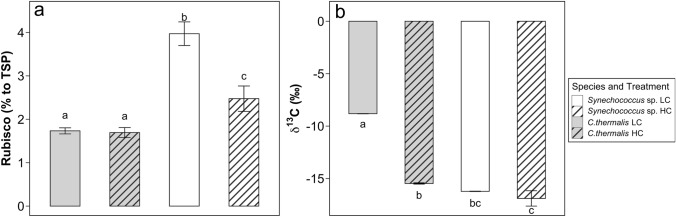
**a** Percentage of Total Soluble Protein (TSP) that corresponds to Rubisco; **b** Cell ^13^C isotopic discrimination (δ^13^C). Values are means ± SD. White color corresponds to *Chroococcidiopsis thermalis* KOMAREK 1964/111 and grey color corresponds to *Synechococcus* sp. PCC6301. The line pattern refers to 2.5% CO_2_—enriched air grown cells (HC) and the empty pattern to ambient air grown cells (0.04% CO_2_, LC). Different letters denote significant differences among strains and CO_2_ treatments (*P* < 0.05, two-way ANOVA followed by Tukey’s test or Kruskal–Wallis test followed with Bonferroni correction for non-parametric data). 3 replicates were used to calculate the % of Rubisco to TSP and 4–7 replicates to calculate δ^13^C

The percentage of Rubisco per total soluble protein (TSP) was invariable between CO_2_ treatments in *C. thermali*s, averaging 1.7% of TSP (Fig. 4a, Supplementary Fig. 2). On the contrary, the percentage of TSP being Rubisco in *Synechococcus* sp. grown under LC was significantly higher than that under HC (4% and 2.5%, respectively). Overall, 1.5-fold higher values of Rubisco per TSP were observed in *Synechococcus* sp. in comparison with *C. thermalis*, when both species were grown under LC (Fig. 4a).

Carbon isotopic fractionation of the biomass (δ^13^C) was significantly less negative in *C. thermalis* (δ^13^C of − 8.8 ‰) than in *Synechococcus* sp. under LC (− 16.2 ‰, Fig. 4b), suggesting a stronger bicarbonate use in the former. δ^13^C of *Synechococcus* sp. was invariable between the two CO_2_ treatments. On the contrary, δ^13^C of *C.* *thermalis* becomes 1.7-fold more negative when grown under HC relative to the value found under LC, acquiring similar values as in *Synechococcus* sp. (δ^13^C of − 15.47 ‰).

### Rubisco gross assimilation modeling

**Fig. 5:**
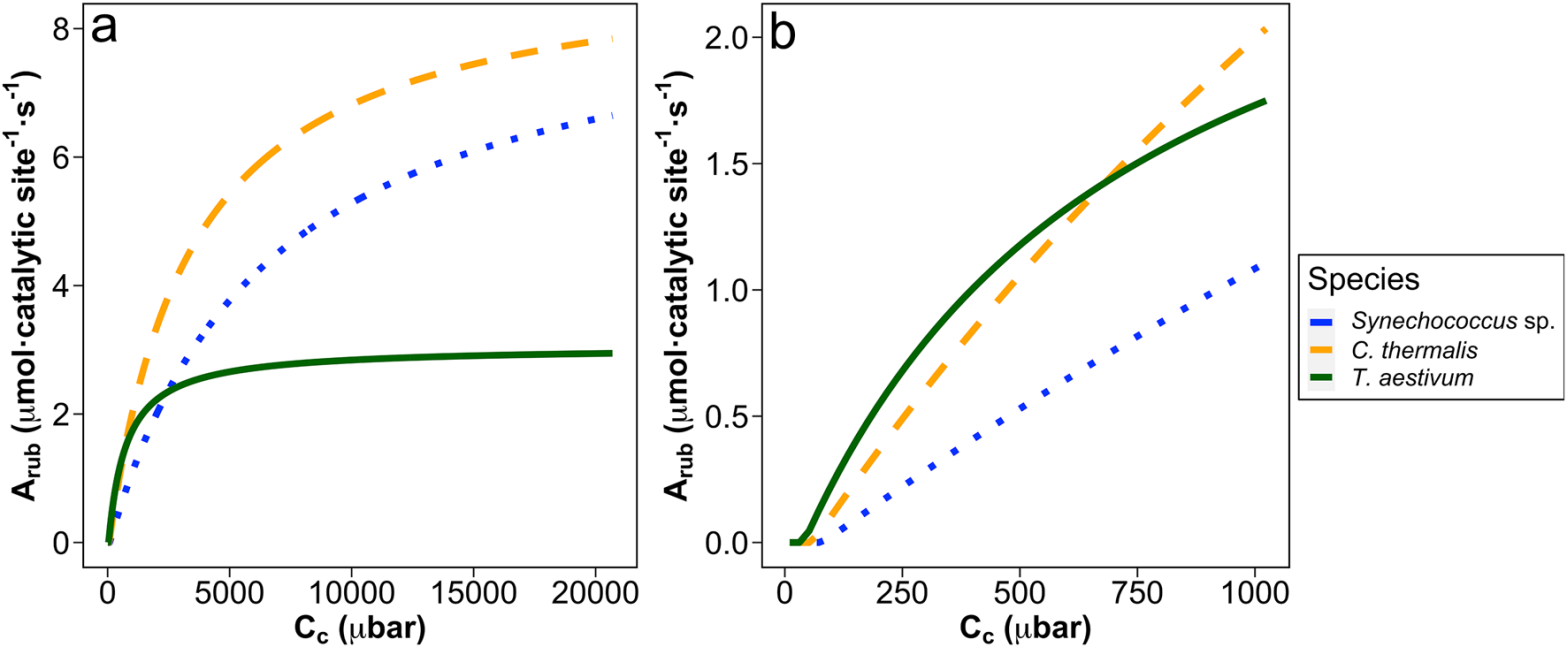
**a** Modeled Rubisco gross assimilation rate (*A*_Rub_) at 25 °C at varying CO_2_ partial pressure at the Rubisco active sites (*C*_c_) of *Synechococcus* sp*.* PCC 6301 (blue dotted line), *Chroococcidiopsis thermalis* KOMAREK 1964/111 (orange dashed line) and *Triticum aestivum* (green line), and **b** Previous graph zoomed in at a *C*_c_ ranging from 0 to 900 µbar

Modeled Rubisco-limited gross assimilation rate (*A*_Rub_) at 25 °C in *C. thermalis* was higher than that of *Synechococcus* sp. at the whole range of CO_2_ partial pressure at the Rubisco active sites (*C*_c_) tested, and higher than *T. aestivum* at *C*_c_ above 700 µbar (Fig. 5a, b). Cyanobacterial *A*_Rub_ saturated at much higher *C*_c_ than *T. aestivum*, but A_Rub_ from *C. thermalis* saturated at lower *C*_c_ than *Synechococcus* sp.

### Anatomical differences between *C. thermalis* and *Synechococcus* sp.

**Fig. 6:**
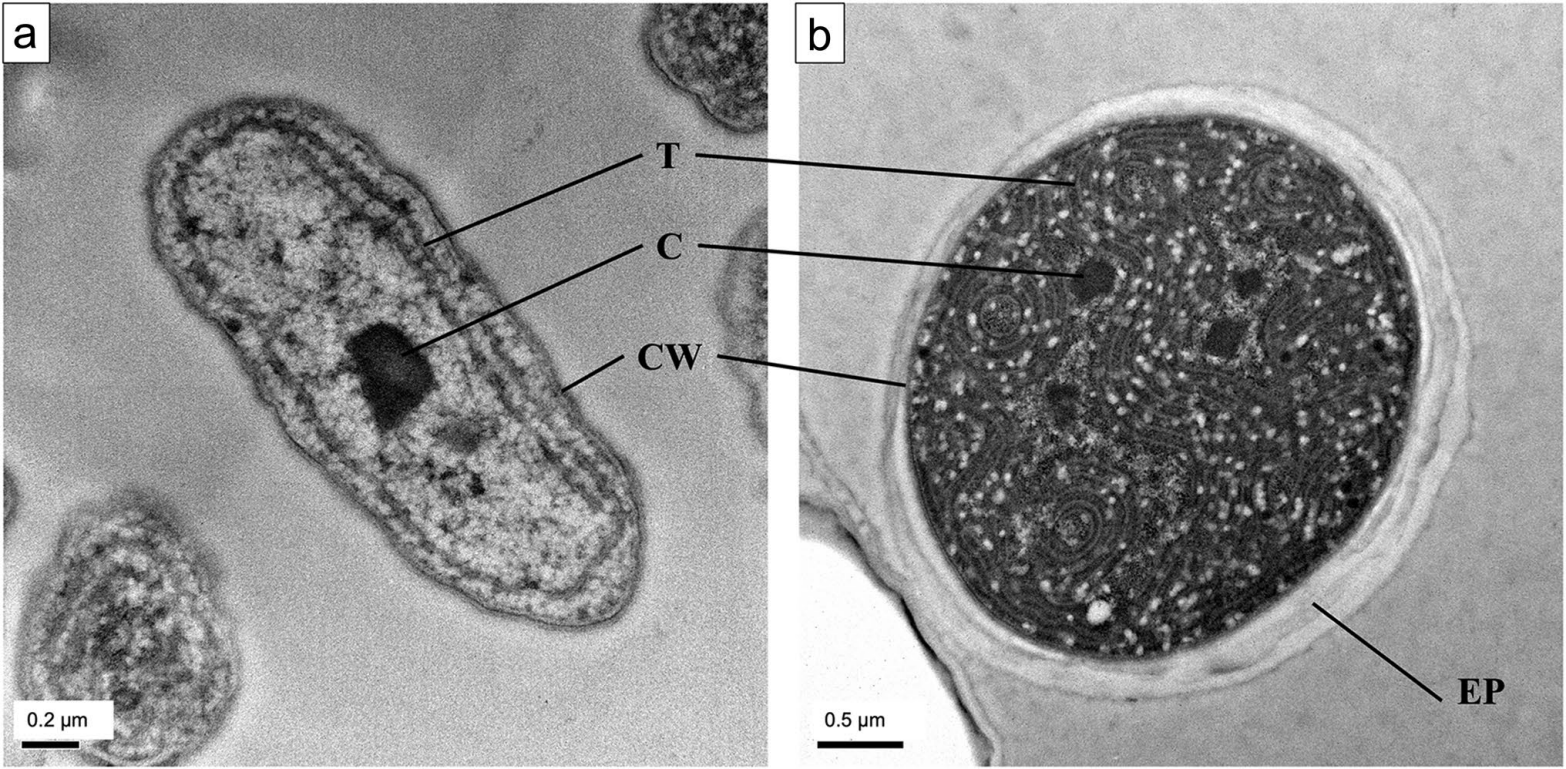
Transmission electron microscope images of **a** *Synechococcus* sp. PCC6301 and **b** *Chroococcidiopsis thermalis* KOMAREK 1964/111. *EP* exopolysaccharide shell, *CW* cell wall, *C* carboxysome, *T* thylakoid membrane. Scale bars are 0.2 µm for *Synechococcus* sp. and 0.5 µm for *C. thermalis*

Cell area (Table 2 and Fig. 6) did not differ between the two CO_2_ treatments in *Synechococcus* sp. (1.6 µm^2^ in LC and 1.7 µm^2^ in HC), while in *C. thermalis*, it was higher in HC-grown cells as compared to LC-grown cells (5.4 µm^2^ and 3.8 µm^2^, respectively). The number of carboxysomes per cell was more than twofold higher in *C. thermalis* than in *Synechococcus* sp. but did not differ between the two CO_2_ treatments within each species (Table 2). The total carboxysome area per cell was independent of the CO_2_ treatment in *Synechococcus* sp., whereas in *C. thermalis,* it was almost double in HC than in LC-grown cells (0.14 and 0.08 µm^2^, respectively). Therefore, the average area for one carboxysome in *C. thermalis* under HC was also twofold higher than under LC. Finally, the percentage of the cell area occupied by carboxysomes (% carboxysome area, Table 2) was 2 to threefold higher in *Synechococcus* sp. than in *C. thermalis*, not being affected by the CO_2_ treatment in any of the species.

**Table 2:** Transmission electron microscopy image characterization of cell area, number of carboxysomes per cell, total carboxysome area per cell, average area of each carboxysome and percentage of the cell area occupied by carboxysomes, in *Synechococcus* sp*.* PCC6301 and *Chroococcidiopsis thermalis* KOMAREK 1964/111 grown either under ambient air (0.04% CO_2_, LC) or 2.5% CO_2_—enriched air (HC)

Species	CO_2_ treatment	Cell area (µm^2^)	No. carboxysomes per cell	Total carboxysome area per cell (µm^2^)	Average area of each carboxysome (µm^2^)	% carboxysome area
*Synechococcus* sp.	LC	1.6 ± 0.4 a	1.3 ± 0.5 a	0.09 ± 0.04 a	0.08 ± 0.04 a	6.1 ± 3.3 a
*Synechococcus* sp.	HC	1.7 ± 0.4 a	1.1 ± 0.4 a	0.08 ± 0.04 a	0.07 ± 0.04 a	4.7 ± 2.8 a
*C. thermalis*	LC	3.8 ± 1.2 b	2.9 ± 1.1 b	0.08 ± 0.03 a	0.03 ± 0.01 b	2.3 ± 1.0 b
*C. thermalis*	HC	5.4 ± 1.6 c	2.5 ± 1.1 b	0.14 ± 0.08 b	0.06 ± 0.02 a	2.7 ± 1.2 b

Values are means ± standard deviations of 30 measured cells per species and treatment. Different letters denote significant differences among different strains and CO_2_ treatments (*P* < 0.05, two-way ANOVA followed by Tukey’s test or Kruskal–Wallis test followed with Bonferroni correction for non-parametric data)

## Discussion

### Remarkable Rubisco kinetic performance in *C. thermalis* within Cyanobacteria

The present results show a large variability in Rubisco kinetics within Cyanobacteria, consistent with what has been shown in recent reviews focused on Rubisco evolution in photosynthetic organisms (Bathellier et al. 2018; Flamholz et al. 2019; Iñiguez et al. 2020). The following message of this finding is that more diverse and efficient Rubiscos can be discovered in barely explored groups, such as Cyanobacteria.

*Chroococcidiopsis thermalis* showed the highest values for Rubisco *S*_c/o_ and carboxylation efficiency ($k_{{{\mathrm{cat}}}}^{{\mathrm{c}}}$/*K*_c_) ever obtained for a cyanobacterium to date. In general, cyanobacterial Rubisco kinetic traits are characterized by low *S*_c/o_ values, along with high $k_{{{\mathrm{cat}}}}^{{\mathrm{c}}}$, *K*_c_ and *K*_o_ (Iñiguez et al. 2020), which suggest that the Rubisco from Cyanobacteria have evolved in an intracellular CO_2_-enriched environment driven by effective CCMs (Flamholz et al. 2019). However, such a high *S*_c/o_ value of *C. thermalis*, which resembles those found in pyrenoid-containing green algae or even those from some vascular plants provided with CCMs (Kubien et al. 2008; Sharwood et al. 2016; Capó-Bauçà et al. 2022b), is the result of a higher affinity for CO_2_ and lower affinity for O_2_ in comparison to other cyanobacterial strains. This suggests that Rubisco evolution in this phylogenetic group is not strictly constrained, and improved *S*_c/o_ is still compatible with high $k_{{{\mathrm{cat}}}}^{{\mathrm{c}}}$ values, as previously discussed by Cummins et al. (2018) and Bouvier et al. (2021). The combination of *C. thermalis* Rubisco kinetic traits could be an adaptative mechanism that allows this species to deal with extremely low environmental CO_2_ concentrations (i.e. at high water temperatures or under desiccation). Moreover, the concentration of Rubisco in *C. thermalis* was insensible to the CO_2_ treatment, contrary to the response observed for *Synechococcus* sp. in our study and for other cyanobacterial strains in other works (Sengupta et al. 2019; Garcia et al. 2021). This indicates that the amount of Rubisco in *C. thermalis* is not involved in the process of acclimation to different environmental CO_2_ concentrations. Hence, Rubisco from *C. thermalis* has evolved towards an enhancement of CO_2_ fixation rates regardless of the environmental CO_2_ concentrations. Indeed, potential Rubisco gross assimilation (*A*_Rub_) from *C. thermalis* exhibited higher values than *Synechococcus* sp. for the whole range of *C*_c_ tested (Fig. 5a).

### Co-evolution of Rubisco and CCMs in *C. thermalis* and *Synechococcus* sp.

There are three main components of the cyanobacterial CCM machinery: CAs, carboxysomes, and inorganic carbon (C_i_) transporters (Badger et al. 2002). The main C_i_ transporters are HCO_3_^−^ transporters and CO_2_ uptake components including NDH-1 dehydrogenase complex (Woodger et al. 2005) whereas CAs can possess an extracellular or intracellular localization. HCO_3_^−^ transporters are involved in the active transport of HCO_3_^−^ through the plasma membrane, while the NDH-1 complex (CO_2_ uptake components) catalyzes the hydration of CO_2_ to HCO_3_^−^ inside the cell (Badger et al. 2002). In this sense, the effects of the inhibitors of CCM components (such as AZ, EZ and DIDS) on the net photosynthetic rates used in the present study for both cyanobacterial strains were similar to those recorded in other studies with mat-forming cyanobacterial strains (Carrasco et al. 2008) and were not reduced by the enriched CO_2_ treatment. However, some differences in the CCM components between the two species were detected, as the anion exchange inhibitor DIDS produced a significantly higher net photosynthetic inhibition in *Synechococcus* sp. than in *C. thermalis* for both CO_2_ treatments. The remarkable high EZ inhibition and the relatively low AZ inhibition of net photosynthesis from *C. thermalis* indicate an important role of internal CAs in its CCM. Therefore, these results suggest that *C. thermalis* might possess an elevated activity of either other type/s of bicarbonate transporter/s not inhibited by DIDS and/or CO_2_ uptake components such as the NDH-1 dehydrogenase complex to supply bicarbonate to the carboxysomes.

Highly effective CCMs were detected in both species since the ratio $K_{{\mathrm{c}}}^{{21{\text{ \% O}}_{2} }} /K_{{\mathrm{m}}}$ _in vivo_ was greater than 2.5, as proposed by Raven et al. (2017) (Fig. 3). However, the CCM effectiveness of *C. thermalis* under LC was among the highest ever reported for a cyanobacterium, concentrating CO_2_ around Rubisco active sites more than 140 times the external CO_2_ levels, 2.3-fold higher than *Synechococcus* sp. PCC6301.

Previous studies with different model cyanobacterial strains reported much lower in vivo photosynthetic affinity for CO_2_ (i.e. higher $K_{\mathrm{m}}\, {\text{in vivo}}$ than *C. thermalis* when grown under similar enriched CO_2_ levels (Whitehead et al. 2014). Thus, *C. thermalis* not only evolved improved Rubisco carboxylation kinetics but also stronger CCMs than other cyanobacterial strains. This response does not follow the inverse relationship between Rubisco carboxylation efficiency and CCM effectiveness previously observed in other photosynthetic groups (Capó-Bauçà et al. 2022a, b) and might indicate that co-evolution between CCMs and Rubisco kinetics in some cyanobacteria is not as constrained as in other phylogenetic groups.

Inhabiting warm-desertic areas, *C. thermalis* has to face extremely high temperatures up to 68ºC (Hindák et al. 2013) that led to a strong limitation in CO_2_ availability (i.e. CO_2_ solubility decreases at higher temperatures). In addition, *C. thermalis* produces a scytonemin rich exopolysaccharide shell to resist desiccation (Vítek et al. 2014; Casero et al. 2021) that might exacerbate CO_2_ limitation through a strong reduction in CO_2_ diffusion from the extracellular medium to Rubisco active sites. Therefore, the development of more effective CCMs in combination with more efficient Rubisco carboxylation kinetics could have contributed to the adaptation of *C. thermalis* to these extreme environments. Since the operation of cyanobacterial CCM depends on the velocity of active HCO_3_^−^ transport and the permeability of the carboxysome to CO_2_ and HCO_3_^−^ (Mangan and Brenner 2014), these processes should be explored in *C. thermalis* in future studies in comparison with model cyanobacterial species to identify the main molecular adaptations that allow this cyanobacterium to possess one of the most effective CCMs ever reported.

In addition, indirect proxies used to detect CCM activity (effect of CCMs inhibitors on net photosynthesis, carbon isotope discrimination, the ratio between the semi-saturation constant for CO_2_ in vitro and in vivo*,* and the morpho-anatomical carboxysome analysis) confirmed the acclimatory capacity of the CCM machinery in *C. thermalis* to respond to changes in environmental CO_2_ concentrations. CCM effectiveness in *C. thermalis* was significantly reduced when grown under HC (since the in vivo photosynthetic semi-saturation constant for CO_2_ was more than threefold higher in HC-grown cells relative to LC-grown cells). This fact, together with a more negative δ^13^C under HC, indicates a downregulation of CCM machinery in *C. thermalis* caused by the increase in environmental CO_2_ concentration. The CCM downregulation could be a mechanism to save energy for other vital processes (Beardall and Giordano 2002) while maintaining similar net photosynthetic rates, as previously observed in other aquatic photosynthetic organisms (Gordillo et al. 2001; Iñiguez et al. 2016; Ma and Wang 2021). Such downregulation was not observed in *Synechococcus* sp., where CCM effectiveness and δ^13^C remained constant independently of the CO_2_ treatment applied, and, as a result, the net photosynthetic rate was almost double under HC despite the decrease in Rubisco quantity per TSP. This might be due to a faster acclimation of *C. thermalis* CCMs than *Synechococcus* sp. CCMs to changes in environmental CO_2_ concentration, which may allow the former to thrive under harsh conditions of high temperature and water scarcity.

### Potential crop yield improvement by the introduction of cyanobacterial carbon utilization mechanisms

Increasing crop yield is a must to face the food needs of the rising population (Ray et al. 2013) since the decreased arable land and climate change impact on crops represent important risks for plant production (Long et al. 2015). Bioengineering approaches for photosynthesis optimization have largely demonstrated the potential for enhancing crop yield, for example by enhancing Rubisco carboxylation capacity (reviewed by Iñiguez et al. (2021)). Several attempts have tried to enhance photosynthetic rates from crops by introducing some basic CCM components and Rubisco from Cyanobacteria. For example, Lin et al. (2014) successfully transformed tobacco plants by expressing *Synechococcus elongatus* PCC 7942 Rubisco with an internal carboxysome protein (CcmM35) producing functional macromolecular complexes. In addition, Long et al. (2018) reconstituted simplified carboxysomes with a minimum set of genes from the genus *Cyanobium* into tobacco chloroplast that were able to encapsulate the cyanobacterial Rubisco. However, none of the transformed tobacco lines expressing simplified carboxysome-like structures were still able to grow equally or faster than wild-type plants. Directions towards fully functional cyanobacterial CCM expression in C_3_ plant chloroplasts go through targeting functional bicarbonate transporter proteins into the chloroplast membranes (Rolland et al. 2016), a hit that might be achievable soon. Here, we have discovered the most CO_2_-specific and efficient cyanobacterial Rubisco ever reported, which represents a potential candidate for bioengineering crop species to increase crop yield in combination with cyanobacterial CCM expression, as the potential capacity for CO_2_ assimilation of *C. thermalis* Rubisco is significantly higher than those from *Synechococcus* sp. PCC6301 and other analyzed cyanobacterial species.

## Conclusions

The present results represent the first complete characterization of inorganic carbon utilization mechanisms in a non-model cyanobacterium, as in vivo CO_2_ assimilation data was complemented with in vitro Rubisco measurements, Rubisco content, ^13^C isotopic discrimination, and the use of CCM inhibitors. This has allowed us to inquire into the inorganic carbon utilization adaptations of a polyextremophilic cyanobacterium. The main highlights of the study are the discovery in *C. thermalis* of the highest values of Rubisco specificity for CO_2_ over O_2_ and catalytic carboxylation efficiency ever obtained for Cyanobacteria, together with the most effective CO_2_-concentrating mechanisms. This allows *C. thermalis* to thrive under CO_2_-limited environments such as elevated temperatures and/or desertic areas. Overall, further exploration of Rubisco kinetics and CCM operation from underrepresented phylogenetic groups is needed to discover highly valuable mechanisms for biotechnological applications.

## Supplementary Information

Below is the link to the electronic supplementary material.

Supplementary file1 (PDF 497 KB)

Supplementary file2 (XLSX 925 KB)
