# Correction to: Singular adaptations in the carbon assimilation mechanism of the polyextremophile cyanobacterium *Chroococcidiopsis thermalis*

**DOI:** 10.1007/s11120-025-01185-y

**Published:** 2026-01-22

**Authors:** Pere Aguiló-Nicolau, Jeroni Galmés, Giacomo Fais, Sebastià Capó-Bauçà, Giacomo Cao, Concepción Íñiguez

**Affiliations:** 1 Research group on Plant Biology under Mediterranean Conditions, Universitat de les Illes Balears, INAGEA, Palma, Balearic Islands Spain; 2 Interdepartmental Centre of Environmental Science and Engineering, University of Cagliari, Cagliari, Italy; 3 Department of Mechanical, Chemical and Materials Engineering, University of Cagliari, Piazza d’Armi, Cagliari, Italy

**Correction to: Photosynthesis Research (2023) 156:231–245**

10.1007/s11120-023-01008-y

The original article has been corrected. In this article Fig. 2 appeared as:

**Fig. 2:**
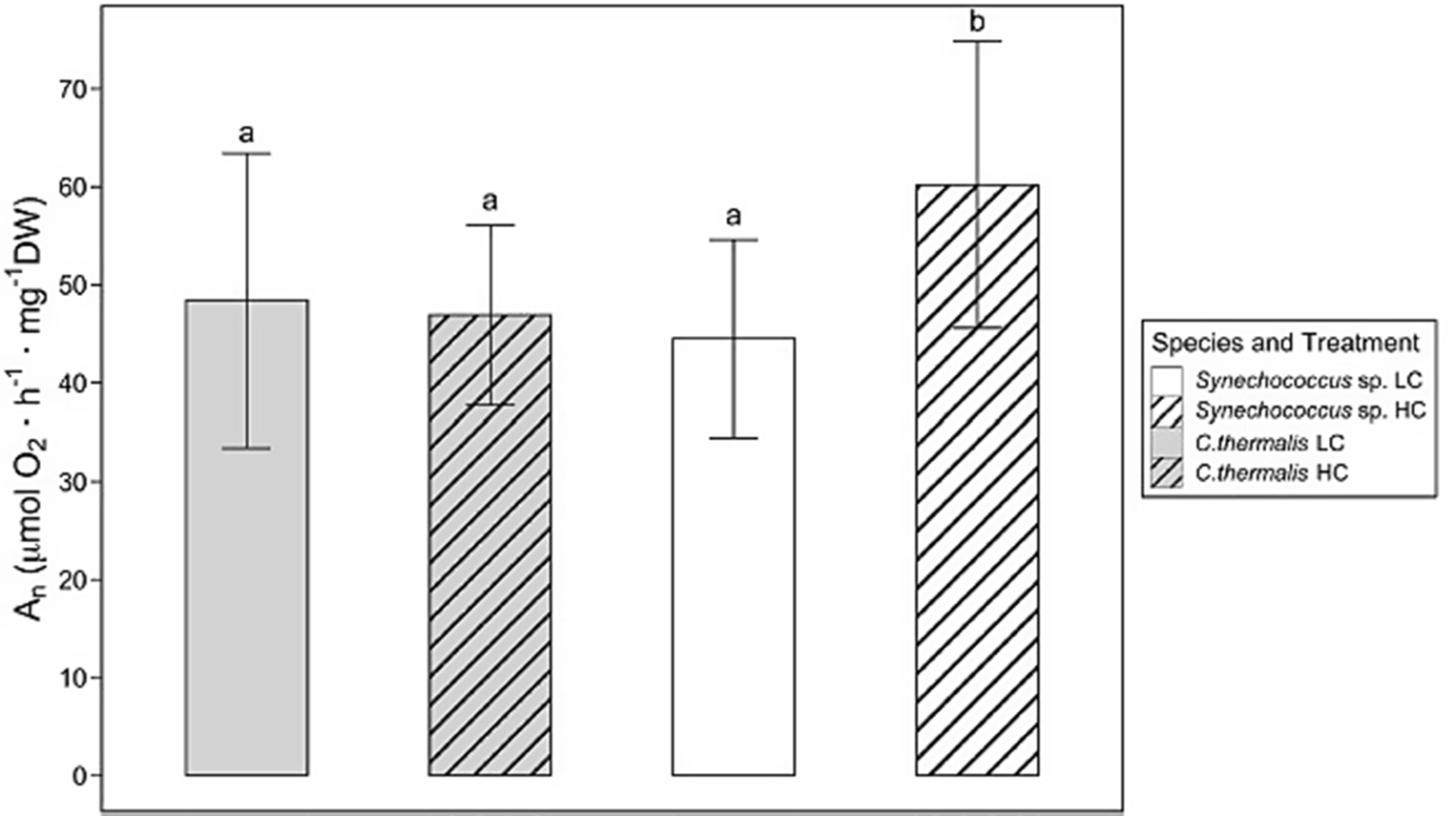
Net photosynthetic rate (An) in *Synechococcus* sp. PCC6301 (white) and *Chroococcidiopsis thermalis* KOMAREK 1964/111 (grey) at 20 °C under ambient air (0.04% CO_2_, LC, empty pattern) or 2.5% CO_2_—enriched air (HC, line pattern) and saturating irradiance (300 µmol photons m^− 2^ s^− 1^). Values are means ± standard deviation of 10 replicates. Different letters denote significant differences among different strains and CO_2_ treatments (*P* < 0.05, two-way ANOVA followed by Tukey’s test or Kruskal-Wallis test followed with Bonferroni correction for non-parametric data)

but should have appeared as:

**Fig. 2:**
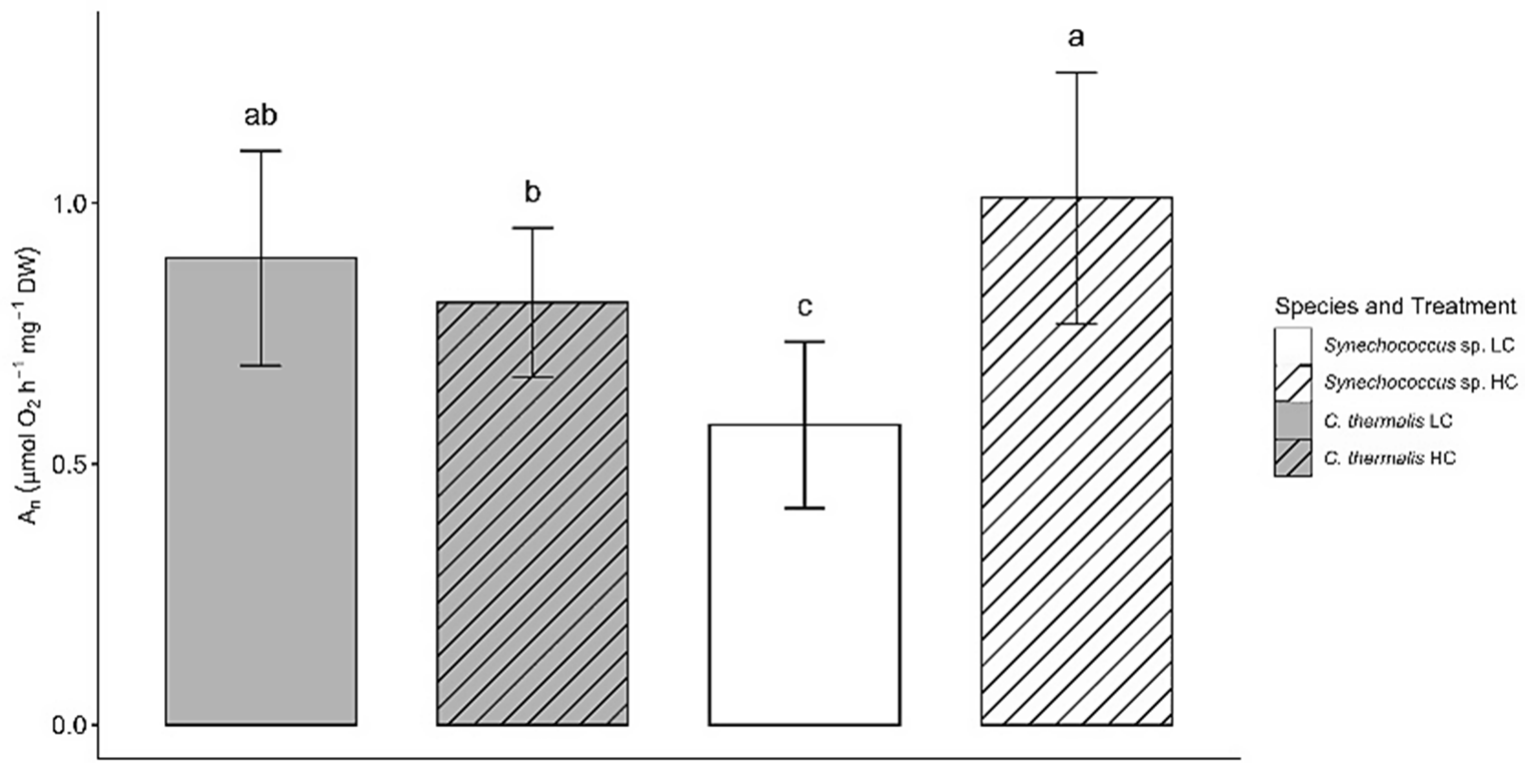
Net photosynthetic rate (An) in *Synechococcus* sp. PCC6301 (white) and *Chroococcidiopsis thermalis* KOMAREK 1964/111 (grey) at 20 °C under ambient air (0.04% CO_2_, LC, empty pattern) or 2.5% CO_2_—enriched air (HC, line pattern) and saturating irradiance (300 µmol photons m^− 2^ s^− 1^). Values are means ± standard deviation of 10 replicates. Different letters denote significant differences among different strains and CO_2_ treatments (*P* < 0.05, two-way ANOVA followed by Tukey’s test or Kruskal-Wallis test followed by Bonferroni correction for non-parametric data)

Correspondingly, the Results section of the paper has also been updated. The paragraph under **Characterization and effectiveness of CO**_**2**_ **concentrating mechanisms in** ***Synechococcus*** **sp. and** ***C. thermalis*** **- Net photosynthesis and effect of CCM inhibitors**.

was originally published as:

Net photosynthetic rate (*An*) from *C. thermalis* and *Synechococcus* sp. under LC did not differ, averaging 47.7 µmol O_2_ h^− 1^ mg^− 1^ DW. On the contrary, *An* from both species under HC (Fig. 2) showed significantly higher values for *Synechococcus* sp. than for *C. thermalis* (60.2 and 46.9 µmol O_2_ h^− 1^ mg^− 1^ DW, respectively). Hence, the *An* of *Synechococcus* sp. under HC was higher than under LC, whereas no differences in *An* of *C. thermalis* were observed between CO_2_ treatments.

and has been corrected to:

Net photosynthetic rate (*A*_n_) in *C. thermalis* was similar under both HC and LC, averaging ~ 0.9 µmol O_2_ h^− 1^ mg^− 1^ DW (Fig. 2). In contrast, *Synechococcus* sp. showed a ~ two-fold higher *A*n under HC than under LC, with *Synechococcus* sp. LC exhibiting the lowest *A*n among all species-treatment combinations.

