# Supplementary material for: Singular adaptations in the carbon assimilation mechanism of the polyextremophile cyanobacterium Chroococcidiopsis thermalis
Source: Photosynth Res. 2023 Mar 20;156(2):231–45. doi: 10.1007/s11120-023-01008-y (PMC10154277; doi:10.1007/s11120-023-01008-y)
Supplement: Supplementary file 1 — Supplementary file1 (PDF 497 KB) [file 11120_2023_1008_MOESM1_ESM.pdf]

Supplementary information

Singular adaptations in the carbon assimilation mechanism of the polyextremophile cyanobacterium *Chroococcidiopsis thermalis*

Photosynthesis research

Aguiló-Nicolau, Pere[1]; Galmés, Jeroni[1*]; Fais, Giacomo[2]; Capó-Bauçà, Sebastià[1]; Cao, Giacomo[2,3]; Iñiguez, Concepción[1].

[1]Research group on Plant Biology under Mediterranean Conditions, Universitat de les Illes Balears, INAGEA, Palma, Balearic Islands, Spain.

[2] Interdepartmental Centre of Environmental Science and Engineering, University of Cagliari, Via San Giorgio 12, 09124, Cagliari, Italy.

[3] Department of Mechanical, Chemical and Materials Engineering, University of Cagliari, Via Marengo 2, 09123, Cagliari, Italy

* Corresponding author: jeroni.galmes@uib.cat

Supplementary Spreadsheet 1. Excel file containing the compiled Rubisco kinetic parameters at 25ºC from Cyanobacteria sorted by strain and Rubisco type, extracted from Iñiguez et al., (2020) and used in Figure 1

Supplementary Table 1. Rubisco kinetic parameters at 25ºC from *Synechococcus* sp. PCC6301 and *Chroococcidiopsis thermalis* KOMAREK 1964/111 shown in Figure 1. Values are means ± standard deviations of 3-6 replicates. Different letters denote significant differences between the two strains ($P < 0.05$, Student's t-test or Mann-Whitney-Wilcoxon test for non-parametric data)

Species	$S_{c/o}$ *(mol · mol⁻¹)*	K_c *(µM)*	$K_c^{21\,\%\,O_2}$ *(µM)*	K_o *(µM)*	k_{cat}^{c} *(s^{-1})*	k_{cat}^{c}/K_c *(s^{-1})*
Synechococcus sp.	48.2 ± 5.8 *a*	147.2 ± 4.6 *a*	215.9 ± 27.7 *a*	631.6 ± 62.6 *a*	8.7 ± 1.2 *a*	0.06 ± 0.01 *a*
C. thermalis	66.0 ± 2.2 *b*	87.3 ± 1.3 *b*	106.9 ± 4.2 *b*	1162.6 ± 182.4 *b*	9.1 ± 0.2 *a*	0.105 ± 0.002 *b*

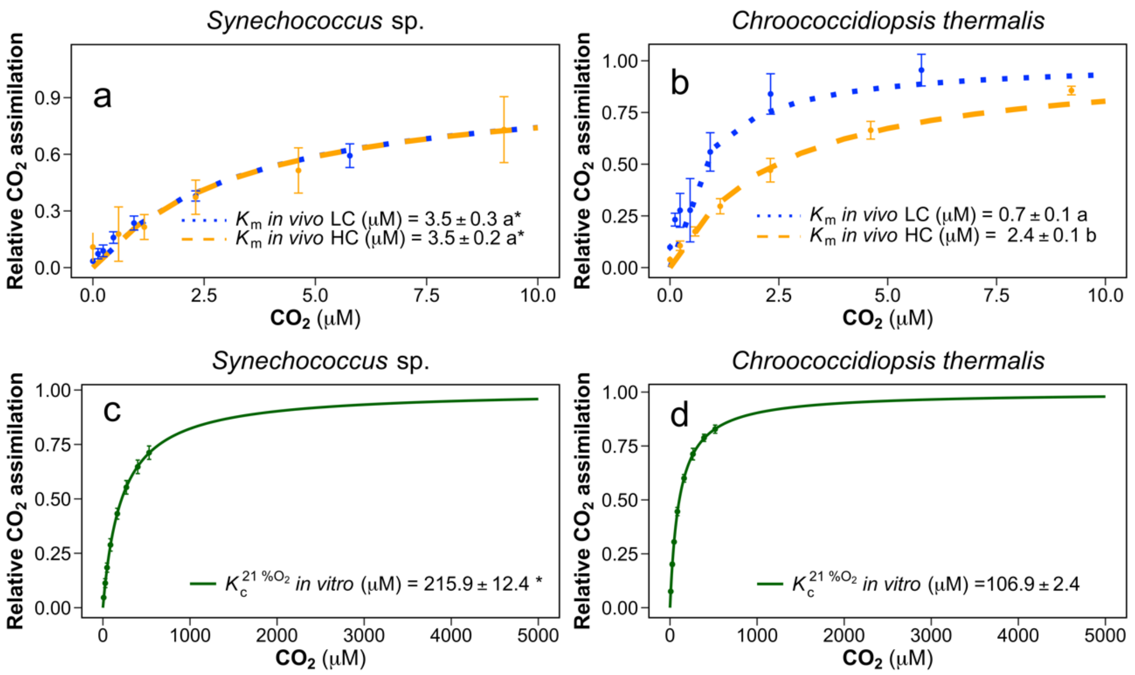

Supplementary Figure 1. Zoom in on Figure 2 with a CO_2 concentration ranging between 0 and 10 μM of photosynthetic *in vivo* CO_2 assimilation of both ambient air grown cells (blue dotted line; LC) and 2.5 % CO_2 grown cells (orange dashed line, HC) from **a**, *Synechococcus* sp. PCC6301 and **b**, *Chroococcidiopsis thermalis* KOMAREK 1964/111. Zoom out of Figure 2 with a CO_2 concentration ranging between 0 and 5000 μM of Rubisco *in vitro* CO_2 assimilation under 21% O_2 (green line) from **c**, *Synechococcus* sp. PCC6301 and **d**, *Chroococcidiopsis thermalis* KOMAREK 1964/111. The maximum Rubisco and photosynthetic CO2 assimilation rates were standardized to 1 in all plots

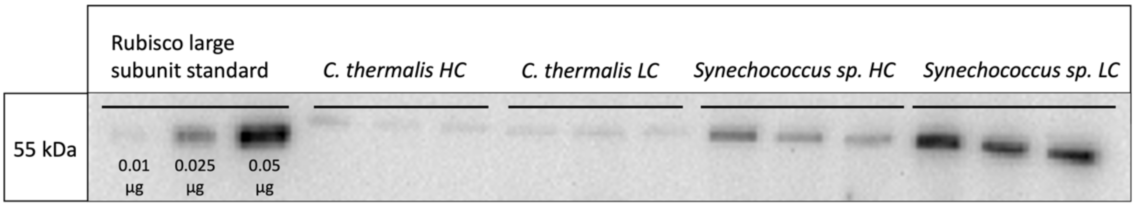

Supplementary Figure 2. Western blot image of Rubisco large subunit from *Chroococcidiopsis thermalis* KOMAREK 1964/111 and *Synechococcus* sp. PCC6301 grown either under ambient air (0.04 % CO_2, LC) or 2.5 % CO_2 – enriched air (HC), accompanied by three different concentrations of Rubisco large subunit standard (AS01017S Agrisera, Sweden). Rubisco large subunit antibody (AS03037 Agrisera, Sweden) was used at 1:20,000 dilution and Goat anti-Rabbit IgG HRP-conjugated secondary antibody (AS09602 Agrisera, Sweden) was used at 1:50,000 dilution
